# Missing Data in OHCA Registries: How Multiple Imputation Methods Affect Research Conclusions—Paper II

**DOI:** 10.3390/jcm15020732

**Published:** 2026-01-16

**Authors:** Stella Jinran Zhan, Seyed Ehsan Saffari, Marcus Eng Hock Ong, Fahad Javaid Siddiqui

**Affiliations:** 1 Centre for Biomedical Data Science, Duke-NUS Medical School, Singapore 169857, Singapore; 2 Department of Emergency Medicine, Singapore General Hospital, Singapore 169608, Singapore; 3 Pre-Hospital & Emergency Research Centre, Duke-NUS Medical School, Singapore 169857, Singapore

**Keywords:** out-of-hospital cardiac arrest, bystander CPR, emergency medical services, missing, multiple imputation

## Abstract

**Background/Objectives:** Missing data in clinical observational studies, such as out-of-hospital cardiac arrest (OHCA) registries, can compromise statistical validity. Single imputation methods are simple alternatives to complete-case analysis (CCA) but do not account for imputation uncertainty. Multiple imputation (MI) is the standard for handling missing-at-random (MAR) data, yet its implementation remains challenging. This study evaluated the performance of MI in association analysis compared with CCA and single imputation methods. **Methods:** Using a simulation framework with real-world Singapore OHCA registry data (N = 13,274 complete cases), we artificially introduced 20%, 30%, and 40% missingness under MAR. MI was implemented using predictive mean matching (PMM), random forest (RF), and classification and regression trees (CART) algorithms, with 5–20 imputations. Performance was assessed based on bias and precision in a logistic regression model evaluating the association between alert issuance and bystander CPR. **Results:** CART outperformed PMM, providing more accurate *β* coefficients and stable CIs across missingness levels. Although K-Nearest Neighbours (KNN) produced similar point estimates, it underestimated imputation uncertainty. PMM showed larger bias, wider and less stable CIs, and in some settings performed similarly to CCA. MI methods produced wider CIs than single imputation, appropriately capturing imputation uncertainty. Increasing the number of imputations had minimal impact on point estimates but modestly narrowed CIs. **Conclusions:** MI performance depends strongly on the chosen algorithm. CART and RF methods offered the most robust and consistent results for OHCA data, whereas PMM may not be optimal and should be selected with caution. MI using tree-based methods (CART/RF) remains the preferred strategy for generating reliable conclusions in OHCA research.

## 1. Introduction

Missing data are a common challenge in all types of studies, particularly in large-scale clinical observational studies, such as those using out-of-hospital cardiac arrest (OHCA) registries. How we handle missingness directly impacts the validity of our analyses and the reliability of study conclusions. Recognising these risks, guidelines, like STROBE [1,2], stress the importance of reporting missing data transparently and explaining the methods used to address it. Despite these recommendations, missing data remain frequently overlooked or inadequately handled in observational OHCA studies.

This study forms part of a planned series of investigations examining methods for handling missing data in OHCA registries. In the first paper of this series (Paper I [3]), we focused on single imputation methods, reflecting their relative simplicity, lower computational demands, and widespread use among clinicians. Using a real-world OHCA dataset, we evaluated a range of statistical and machine learning-based single imputation methods for association analyses. The findings demonstrated that complete-case analysis (CCA), where observations with missing data are simply ignored, was not ideal. It led to more biased results (i.e., the estimated association did not accurately reflect the true relationship between variables) and wider confidence intervals (CIs), meaning the results were less precise. On the other hand, single imputation methods like K-Nearest Neighbours (KNN) and missingness-indicator (MxI) performed better. They helped reduce bias and provide more reliable results, while still being relatively simple to implement. Hence, they can serve as useful alternatives to CCA for drawing more accurate conclusions.

While single imputation methods are appealing because of their simplicity, they do not fully capture the uncertainty caused by missing data, as they only provide one guess for each missing value. This limitation can lead to artificially narrow CIs, and the distribution of imputed values might not align with the original data. Furthermore, although some machine learning single imputation methods, like KNN, can handle some complex relationships, they may still struggle to address more intricate patterns, especially when the missing data are influenced by unobserved factors.

Multiple imputation (MI) offers a more robust solution by explicitly incorporating uncertainty into the imputation process [4]. First developed by Rubin in the late 1970s [5,6,7], MI is now widely used, especially when the missing data are thought to be missing at random (MAR) [8,9]. Rather than filling in just one value for each missing data point, MI generates several plausible sets of imputed values. These complete datasets are then analysed separately and combined to produce final estimates and standard errors that more accurately reflect uncertainty due to missingness. Austin et al. provide an accessible, practical tutorial on MI and show its advantages in clinical research [4].

The most common and flexible MI procedure is the Multiple Imputation by Chained Equations (MICE) algorithm [10]. It allows researchers to customise the imputation process, including which variables to use, the imputation model and how many imputations (m) to generate. This flexibility makes MICE adaptable to various data types and research goals [11], but it also introduces additional methodological complexity, which may limit its routine adoption by clinicians [12,13].

For these reasons, multiple imputation was not examined in detail in Paper I. Including both single and multiple imputation methods in a single study would have limited the depth and clarity of the evaluation of each approach. Instead, Paper I was intentionally designed to establish a consistent simulation framework and to identify the most promising single imputation approach, providing a foundation for a more focused assessment of MI in this second study.

While MI is increasingly supported by many software tools, there is limited practical guidance for clinicians, and few studies have compared different MI configurations using a real-world dataset like those in OHCA. As a result, many clinicians still rely on simpler methods, even though MI could provide more accurate results. Key questions, therefore, remain, such as the different MI configurations and how MI compares with simpler methods like KNN and MxI. These are important considerations for the OHCA research community.

Building directly on the framework and findings of Paper I [3], the objective of this article (Paper II) is to evaluate the performance of MI under different configurations (e.g., imputation models and number of imputations). Using the same real-world OHCA dataset and simulation design, we compare MI with CCA and the top-performing single imputation method identified previously (KNN). Through this comprehensive simulation-based evaluation, we aim to offer practical guidance for OHCA researchers and clinicians on selecting appropriate missing data strategies while balancing statistical accuracy and computational feasibility.

## 2. Materials and Methods

### 2.1. Study Design and Setting

Data were obtained from two prospectively maintained registries: the Pan-Asian Resuscitation Outcomes Study (PAROS) [14] and the Singapore Community First Responder system, myResponder [15]. The study included adult OHCA cases (≥18 years) that could have triggered myResponder alerts between 1 January 2016 and 31 December 2020. We excluded cases that were witnessed by emergency medical services (EMS) and those with missing values for call time or first rhythm. These inclusion and exclusion criteria are identical to those employed previously to ensure comparability across the study series.

The analysis of interest is the association between alert issuance (binary exposure) and bystander CPR (BCPR, binary outcome), adjusted for potential confounders, including age, gender, witness type, arrest location, call time, and first rhythm.

Following the simulation framework described in Paper I [3], we used the same reference dataset to generate 1000 artificial datasets under MAR missingness at 20%, 30%, and 40%, reflecting increasing levels of missing data severity and forming the basis of subsequent sensitivity analyses. Missing covariates were age, witness type, and call time. The same seed number was used for reproducibility purposes. This methodological setup allows Paper II to evaluate the performance of MI methods under conditions identical to those used previously. Further details on the cohort and the creation of missing data are provided in Paper I [3].

### 2.2. Methods to Handle Missing Data

**Figure 1:**
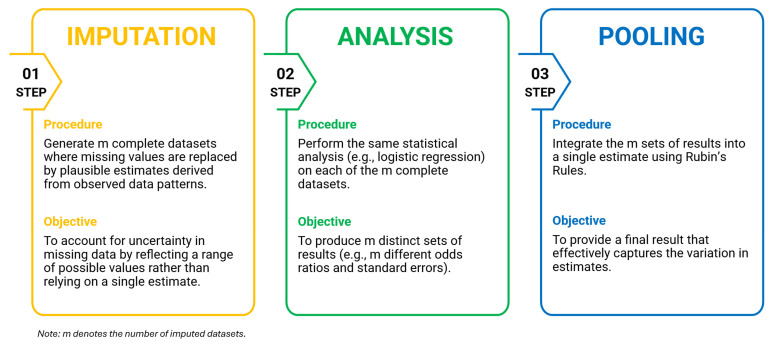
Multiple imputation process flowchart using out-of-hospital cardiac arrest registry data for Singapore (January 2006–December 2020).

This paper, building on previous work, extends that investigation by directly comparing the single imputation approaches with a suite of MI methods. Unlike single imputation, which replaces each missing value with a single value, MI generates several plausible, but different, complete datasets to account for the uncertainty inherent in missing data. This iterative process involves three steps: imputation, analysis, and pooling (Figure 1).

The most widely used MI algorithm, MICE, imputes missing values sequentially on a variable-by-variable basis. For each variable with missing data, a separate imputation model can be specified based on the variable type. MICE can be implemented in R using the *mice()* function from the *mice* package [10]. The selection of an appropriate imputation method and the number of imputations (m) are important inputs for the validity and performance of MI. The model specifies the relationships between the observed and missing variables, guiding how the missing values are estimated. The default imputation methods in *mice()* are predictive mean matching (PMM) for continuous variables, logistic regression for dichotomous variables, and polynomial regression for unordered categorical variables. In this study, we focused on a set of commonly used imputation methods available in *mice()* that are suitable for any type of data:

- Predictive mean matching (PMM): A non-parametric imputation method similar to regression, designed to preserve the original distribution and avoid impossible or unrealistic values. For each missing value, PMM first uses a regression model to predict a value. It then randomly selects the final imputed value from the actual observed value of the cases with predictions most similar to the predicted value for that missing case [16].
- Classification and regression trees (CART): A non-parametric tree-based ML method that eliminates the need for a predefined imputation model. It predicts missing values by recursively partitioning the data through a series of binary splits. These splits are determined by finding optimal predictor thresholds that divide the sample into more homogeneous subgroups. For each missing value, it uses the rules from the tree to assign a plausible value. This method remains stable in the presence of outliers, multicollinearity, and skewed distributions, and can also incorporate interactions and non-linear relationships [17].
- Random forest (RF): A non-parametric tree-based ML imputation method that builds upon the concept of CART. It introduces controlled variation via bootstrapping to create an extensive collection of distinct regression trees. For a given missing value, the RF model generates potential imputed values from all the individual trees in the forest. The final imputation is a random draw from this set of values. It shares the same robustness and flexibility advantages as CART while minimising the risk of overfitting individual trees [18].

By incorporating machine learning-based imputation methods, the analysis captures a broad spectrum of modelling assumptions, including non-linearities and interaction effects, thereby strengthening the robustness of the comparative evaluation.

### 2.3. Statistical Analysis

This study was designed as a formal simulation-based evaluation of imputation methods, following established methodological guidance for simulation studies. By repeatedly generating datasets with controlled missingness mechanisms and known ground truth, this framework allows for rigorous assessment of estimator accuracy and robustness across a range of realistic scenarios.

To evaluate the performance of the various imputation approaches, we used the reference dataset (containing no missing values) as our population of interest. On this reference data, we established the “true” association by fitting a multivariable logistic regression model. This analysis estimated the relationship between alert issuance and BCPR, while controlling for key covariates: age, gender, witness type, call time, arrest location, and first rhythm. Logistic regression was intentionally selected as the target inferential model, as it reflects standard analytic practice in OHCA research for binary outcomes and allows for direct evaluation of how missing data handling impacts clinically interpretable effect estimates.

We maintained the simulation framework described in our previous work, which involved creating 1000 amputed datasets (i.e., artificially introducing missingness), applying the imputation methods and conducting the same association analysis on these imputed datasets. The performance of each imputation method was assessed using the following metrics: bias (deviation of $\hat{\beta }$ estimates from the true value), empirical SE (variability of $\hat{\beta }$ estimates across repetitions), and nominal coverage of 95% CI (proportion of CIs containing the true value). We also assessed model-specific metrics, including the root mean squared error (RMSE) and the Akaike Information Criterion (AIC). The use of 1000 simulation replications per scenario ensures stable estimation of bias, coverage, and variability, and is consistent with commonly recommended practice for simulation-based methodological studies. The distribution of these performance metrics was analysed to assess variability across simulations and comparability with the reference values. Supplementary Figure S1 in Paper I [3] illustrates the flowchart of the simulation process.

Sensitivity analyses were conducted by varying the proportion of missing data (20%, 30%, and 40%), reflecting mild to substantial missingness, to assess the robustness of imputation performance, estimator accuracy, and inferential validity as the extent of missing data increased. Furthermore, the simulation was repeated using different numbers of imputations (m = 5, 10, 15, 20) to assess the stability of estimates under differing levels of Monte Carlo error. We investigated its influence on the method performance, as according to recent studies, the number of imputations may align with the missingness proportion [7,12]. We also compared the computational time for each imputation method. Since MICE is an iterative procedure, we monitored convergence via trace plots on a random subset of the 1000 iterations. We examined the plots for the mean and standard deviation of the imputed values, looking for lines that overlapped randomly without long-term trends. These patterns confirmed that no convergence issues occurred. All the statistical analyses were performed using R software version 4.3.2 and RStudio version 2024.09.0+375 on a computer equipped with an Intel Core i7-1365U, 32 GB of RAM, and running Windows 11 Education, Version 24H2.

## 3. Results

Patient and OHCA event characteristics for the entire cohort (N = 13,274) were previously detailed in Table 1 of Paper I [3]. Here, we focus on the performance of the MI methods with PMM, RF, and CART imputation in the multivariable logistic regression analysis of BCPR. These approaches were compared against CCA and the single imputation methods, particularly the top-performing KNN.

**Figure 2:**
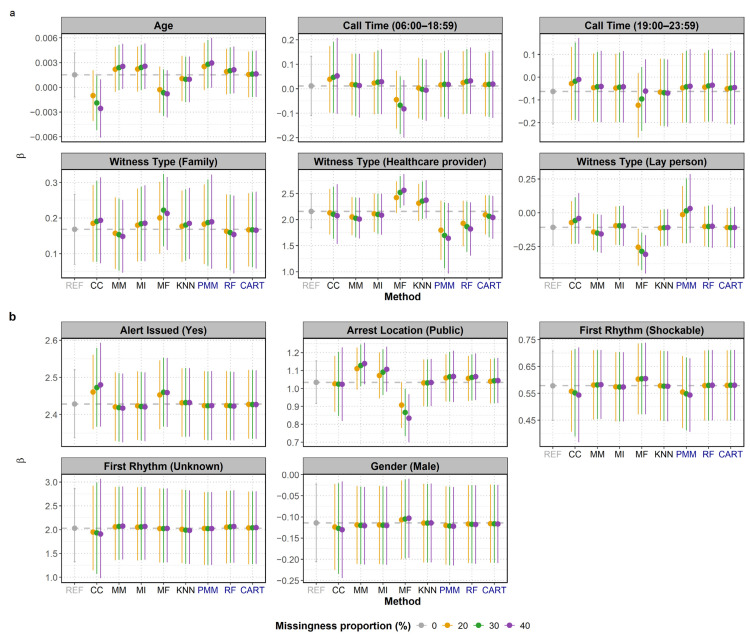
Mean *β* coefficients and 95% confidence intervals (CIs) for the effect of each covariate on BCPR, based on 1000 simulations, using single and multiple imputation methods: (**a**) for covariate with missing values and (**b**) for covariate with non-missing values. Results for missing data proportions of 20%, 30%, and 40% are shown in orange, green, and purple, respectively. The dashed grey line represents the “true” coefficient from the reference dataset with no missing data. REF—no missing data (reference); CC—complete-case; MxI—missingness-indicator; MF—missForest; KNN—K-nearest neighbours; PMM—predictive mean matching; RF—random forest; CART—classification and regression trees.

The mean *β* coefficients and their 95% CIs resulting from the various imputation methods were compared against the reference (REF) values to assess bias and uncertainty. As shown in Figure 2, all methods produced varying degrees of proximity to the reference lines, though the closeness varied across covariates and missingness proportions. Crucially, none of the MI methods altered the statistical significance of the results compared to the reference (i.e., whether the CI consistently included zero). However, the lower bound of the CI for PMM’s estimate of age approached the zero line at 30% and 40% missingness, making the result visibly borderline.

The performance was not uniform across covariates with missing values, like age, witness type, and arrest location (public), which exhibited more noticeable shifts or wider intervals. CART generally offered the closest *β* coefficient estimate to the reference and maintained the most stable CIs among the MI methods across all covariates, suggesting it is the most robust MI approach in this analysis. RF also provided mean *β* coefficients in close agreement with the reference, but its stability was slightly lower than CART, particularly for covariates with missing data (Figure 2a). PMM showed the largest deviation from the reference line and the widest 95% CIs, indicating higher uncertainty and bias relative to the other MI methods. The mean β coefficient for the first rhythm (shockable) was similar to CCA.

The 95% CIs of the MI methods generally overlapped with the reference *β* coefficient, particularly at lower missingness proportions. This confirmed their ability to capture the true association. As expected, MI methods incorporated uncertainty around the imputed values, which resulted in wider CIs compared to the single imputation methods. This highlights the tendency of single imputation methods to underestimate the true variability, leading to overly narrow CIs and potentially inflated risk of a type I error (i.e., they are more likely to exclude zero when no true association exists, causing the researcher to falsely conclude that a variable is statistically significant).

Supplementary Figure S1 visually summarises the bias across the 1000 iterations for all covariates, showing the difference between the estimated $\hat{\beta }$ and the true *β*. The MI methods (PMM, RF, and CART) exhibited a median bias closer to zero compared to CCA and most SI methods. While the variability (box width) of bias increased for all methods at higher missingness proportions, it remained smaller for MI compared to SI methods.

Among the single imputation methods, KNN often maintained the closest alignment to the reference and produced results similar to the MI methods, demonstrating a consistent ability to preserve the mean *β* coefficients. Overall, MI with CART imputation offered the closest $\hat{\beta }$ coefficient estimate to the reference and reasonable confidence intervals.

**Figure 3:**
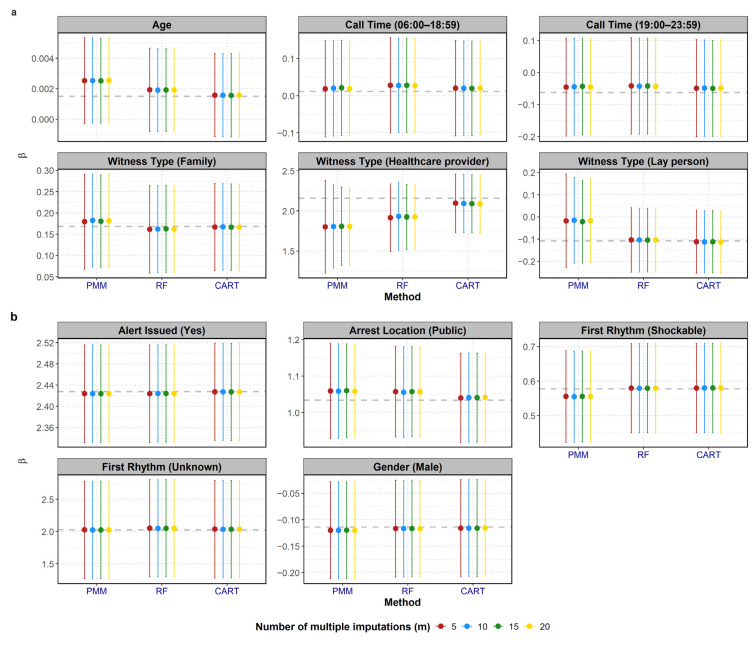
Mean *β* coefficients and 95% confidence intervals (CIs) for the effect of each covariate on BCPR, based on 100 simulations with 20% missingness proportion, for different numbers of multiple imputations (m): (**a**) for covariate with missing values and (**b**) for covariate with non-missing values. Results for m values of 5, 10, 15, and 20 are shown in red, blue, green, and yellow, respectively. The dashed grey line represents the “true” coefficient from the reference dataset with no missing data. PMM—predictive mean matching; RF—random forest; CART—classification and regression trees.

We also investigated the influence of the number of imputations on the performance of MI methods. Figure 3 presents the mean *β* coefficients and 95% CIs across all covariates at a fixed missingness proportion of 20%, when varying m at 5, 10, 15, and 20. Overall, the influence of m was minimal compared to the influence of the imputation methods. The point estimates from the three methods (PMM, RF, and CART) consistently clustered together across the different values of m. For a fixed m, the relative performance of the methods remained consistent: CART was the closest to the reference line, while PMM showed the largest deviation among the three MI methods. While the point estimates were stable, the 95% CIs appeared to narrow slightly as the number of imputations increased from 5 to 20. This is most visually apparent in the PMM results for witness type (healthcare provider) and witness type (lay person), where the difference between the m = 5 CIs and the m = 20 CIs was most noticeable.

The median RMSE across simulations for the MI methods was higher than for the single imputation ones, but their overall variability was smaller compared with the ML-based single imputation approaches. The MI methods produced AIC values close to the reference AIC, indicating a good recovery of the overall analytical model fit. Specifically, CART achieved the lowest AIC median, outperforming PMM, which showed a minor increase in AIC with higher missingness (Supplementary Figures S4 and S5). Furthermore, all three methods presented smaller empirical SEs across all variables and good coverage of the 95% CIs. CART proved to be the most robust MI method in maintaining coverage, whereas PMM and RF showed some minor reduction in coverage for the witness type (healthcare provider) covariate (Supplementary Table S1).

## 4. Discussion

This study used a simulation framework based on real-world Singapore OHCA data (PAROS and myResponder) to systematically evaluate the performance of CCA, common single imputation approaches, and MI methods in estimating associations from a multivariable logistic regression model. By comparing the resulting *β* coefficients and confidence intervals with a reference dataset without missingness, our findings provide practical and methodological guidance for clinicians conducting OHCA research.

Paper I [3] established the performance of CCA and various single imputation methods. As noted previously, CCA generally suffered from wider confidence intervals and greater potential for bias, especially when data are MAR. Among single imputation methods, KNN demonstrated the most consistent ability to preserve mean *β* coefficients and reasonable uncertainty, aligning closely with reference values across missingness proportions. Its performance was often comparable to the MI method with CART, suggesting that KNN can serve as a pragmatic option when computational resources or methodological expertise are limited.

However, all single imputation methods underestimate uncertainty because they treat imputed values as if they were observed, as reflected by the narrower CIs of the investigated single imputation methods. Multiple imputation is widely recommended for handling MAR missingness because it incorporates uncertainty around the imputed values through Rubin’s rule. As seen in Figure 2, MI methods produced wider, more realistic confidence intervals and maintained correct statistical significance for all covariates compared with the reference dataset. Among the MI methods, CART emerged as the most robust, producing *β* coefficients closest to the reference and stable confidence intervals across covariates. RF also performed well but showed slightly less stability, especially for covariates with missing data. In contrast, PMM, despite being the default imputation method for continuous variables, demonstrated the greatest deviation from reference values and the widest CIs, approaching CCA performance for some covariates. PMM relies on observed cases in the dataset that look similar to the case with missing data; therefore, they might perform poorly when there are not enough similar cases in the original dataset. Tree-based methods (CART and RF) should be preferred when nonlinear relationships exist in the data, as they are able to naturally capture that.

The observed stability of the mean *β* coefficients across the different numbers of imputations is consistent with the literature showing that a small number of imputations is generally sufficient to obtain a valid estimation of point estimates. In our study, increasing m produced negligible effects on *β* estimates. However, confidence intervals narrowed slightly as m increased, particularly in PMM, reflecting reduced simulation error.

Several authors proposed how to choose the m required, with a simple rule-of-thumb being the number of imputations to be greater than or equal to the missingness proportion [7,12,19,20,21]. A more sophisticated rule for proper standard error estimates can be implemented using the *howManyImputations* R package [21]. The *mice()* function uses 5 as the default, which can be used as a starting point [12]. Higher values of m are recommended to obtain more accurate standard errors. The ideal m should be selected such that the resulting pooled estimates do not differ much across repetitions of the multiple imputation.

Although MI provides statistically valid inference, it also presents challenges that may hold back adoption in clinical research. Firstly, implementing MI, especially for large datasets or complex imputation models, can be computationally intensive and time-consuming. Supplementary Figure S3a shows that the ML-based approaches (MF, KNN, RF, and CART) were the most computationally expensive, with RF requiring the longest elapsed time, followed by MF. This is expected given its need to build numerous decision trees. Advanced single imputation methods like KNN also required significant time, with MF surpassing MI methods when the number of imputations is low (m = 5). Furthermore, Figure S3b shows that computation time for all MI methods increases with the number of imputations, with RF having the highest cost per imputation and PMM remaining the fastest MI method. However, given a reasonable size of dataset, the resulting difference in computational time between MI and SI methods may be negligible, and easily outweighed by the statistical benefits of obtaining unbiased estimates and correctly specified uncertainty.

As a second challenge, selecting and correctly specifying the appropriate imputation model for each variable (e.g., handling interactions, non-linear relationships, or different variable types) is important to avoid biased results [22]. Thirdly, diagnostic checks are needed to ensure that the imputation algorithm has converged, that the MAR assumption is reasonable, and that the imputed values follow the expected variable distributions. All these steps demand technical expertise that can be difficult to carry out without professional training.

Despite its advantages, MI is not universally necessary or optimal for every analysis. MI adds complexity relative to KNN or MxI. These simpler single imputation methods may offer reasonable preliminary estimates when uncertainty quantification is not the primary objective. However, when single imputation is used, its fundamental statistical limitations must be clearly acknowledged. SI methods (including advanced options like missForest, which is popular in clinical research) produce a single complete dataset, failing to account for the uncertainty caused by the missing data. As a consequence, this limitation leads to two critical problems that scale with the amount of missingness: confidence intervals become falsely narrowed, thus underestimating uncertainty, and the associations between variables appear exaggerated, potentially leading to an inflated risk of false positives [23].

The selection of an imputation method is not only a statistical decision, but also a fundamental step that directly influences the reliability of pre-hospital research findings. The challenging conditions at the scene of cardiac arrest often make it hard to record important details, such as adrenaline administration, alternative medications, other pre-hospital interventions, response time, and location of arrest. Because these factors are primary determinants of patient outcomes, the consequences of mishandling their missingness can be magnified, potentially leading to erroneous clinical conclusions. This is particularly relevant for the COVID-19 pandemic period, which disrupted standard care and data collection protocols [24]. While our simulation used a core set of variables, our results show that robust imputation methods, like MI using CART, are necessary to account for the complex missingness patterns and relationships between variables that arise during such periods.

### Limitations and Future Perspectives

While this study provides a robust evaluation of imputation methods, several limitations should be acknowledged. First, our results were based on simulations from a single national OHCA database. Although the dataset is large and representative, external validation in other clinical contexts and patient populations is necessary to confirm the generalizability of our findings. Second, we focused primarily on the MAR mechanism; missingness occurring under other assumptions, such as missing-not-at-random, might require different approaches. Finally, we did not include emerging deep learning-based imputation methods. These approaches have demonstrated potential to outperform traditional approaches; however, their practical use remains limited by substantial computational demands and challenges related to portability and interpretability [25,26,27,28].

To address the challenges posed by missing data, future work should focus not only on methodological rigour but also on the data collection process itself. Strategies to minimise missingness and manual entry errors at the source include the automation of data synchronisation between dispatch centres, ambulance logs, and electronic health records. Furthermore, implementing real-time data validation within frontline digital tools could ensure that critical variables (e.g., timestamps and witness status) are captured accurately at the point of entry. By reducing baseline missingness, such improvements in registry design would complement advanced imputation methods and facilitate more reliable cross-border research.

Ultimately, to bridge the gap between methodological advances and clinical practice, our follow-up work will provide a general framework for clinical use and develop user-friendly software tools. By simplifying the implementation of advanced imputation methods, we hope to promote the adoption of best practices within the OHCA research community. This will ensure that clinical conclusions are based on the most robust and reliable data possible, improving the evidence based for emergency cardiac care.

## 5. Conclusions

This study demonstrates that the choice of imputation method significantly impacts the validity of association analyses in OHCA research. While CCA and single imputation methods like KNN are pragmatic for preliminary exploration, they fail to adequately account for the uncertainty of missing data. MI using CART provided the most robust and stable performance across all simulation scenarios, balancing point estimate accuracy with realistic uncertainty quantification. Its effectiveness highly depends on the selection of an appropriate imputation method. We recommend that clinical researchers adopt MI as the standard for primary analyses and transparently report the specific imputation methods and diagnostic steps used to ensure that emergency care evidence is built upon a reliable statistical foundation.

## Data Availability

The raw data supporting the conclusions of this article will be made available by the authors on request.

## Supplementary Materials

The following supporting information can be downloaded at: https://www.mdpi.com/article/10.3390/jcm15020732/s1. Figure S1: Bias for each coefficient in the multivariable logistic regression model predicting BCPR; Figure S2: Bias for each coefficient in the multivariable logistic regression model predicting BCPR; Figure S3: Computational time (seconds) for various imputation methods using a randomly selected amputed dataset: (a) imputation time for single imputation methods (CC, MM, MxI, MF, KNN) and multiple imputation methods (PMM, RF, CART) with a fixed number of imputations (m = 5); Figure S4: RMSE for each model across 1000 simulations grouped by method and coloured by proportion of missing data; Figure S5: AIC for each model across 1000 simulations grouped by method and coloured by proportion of missing data; Table S1: Multivariable logistic regression analysis of probability of receiving BCPR. Results are derived from 1000 simulated datasets based on the OHCA reference dataset (N = 13,274), where missingness was artificially introduced at different proportions (20%, 30% and 40%).

## Abbreviations

The following abbreviations are used in this manuscript:

AIC	Akaike Information Criterion
BCPR	Bystander cardiopulmonary resuscitation
CART	Classification and Regression Trees
CC	Complete-case
CCA	Complete-case analysis
CFR	Community first responder
CI	Confidence interval
KNN	K-nearest neighbours
MAR	Missing-at-random
MF	missForest
MI	Multiple imputation
MICE	Multiple Imputation by Chained Equations
ML	Machine learning
MxI	Missingness-indicator
OHCA	Out-of-hospital cardiac arrest
PAROS	Pan-Asian Resuscitation Outcomes Study
PMM	Predictive mean matching
RF	Random forest
RMSE	Root mean squared error
SI	Single imputation
