# Supplementary material for: Missing Data in OHCA Registries: How Multiple Imputation Methods Affect Research Conclusions—Paper II
Source: J Clin Med. 2026 Jan 16;15(2):732. doi: 10.3390/jcm15020732 (PMC12842346; doi:10.3390/jcm15020732)
Supplement: Supplementary file 1 [file jcm-15-00732-s001.zip › jcm-4077774-supplementary.pdf]

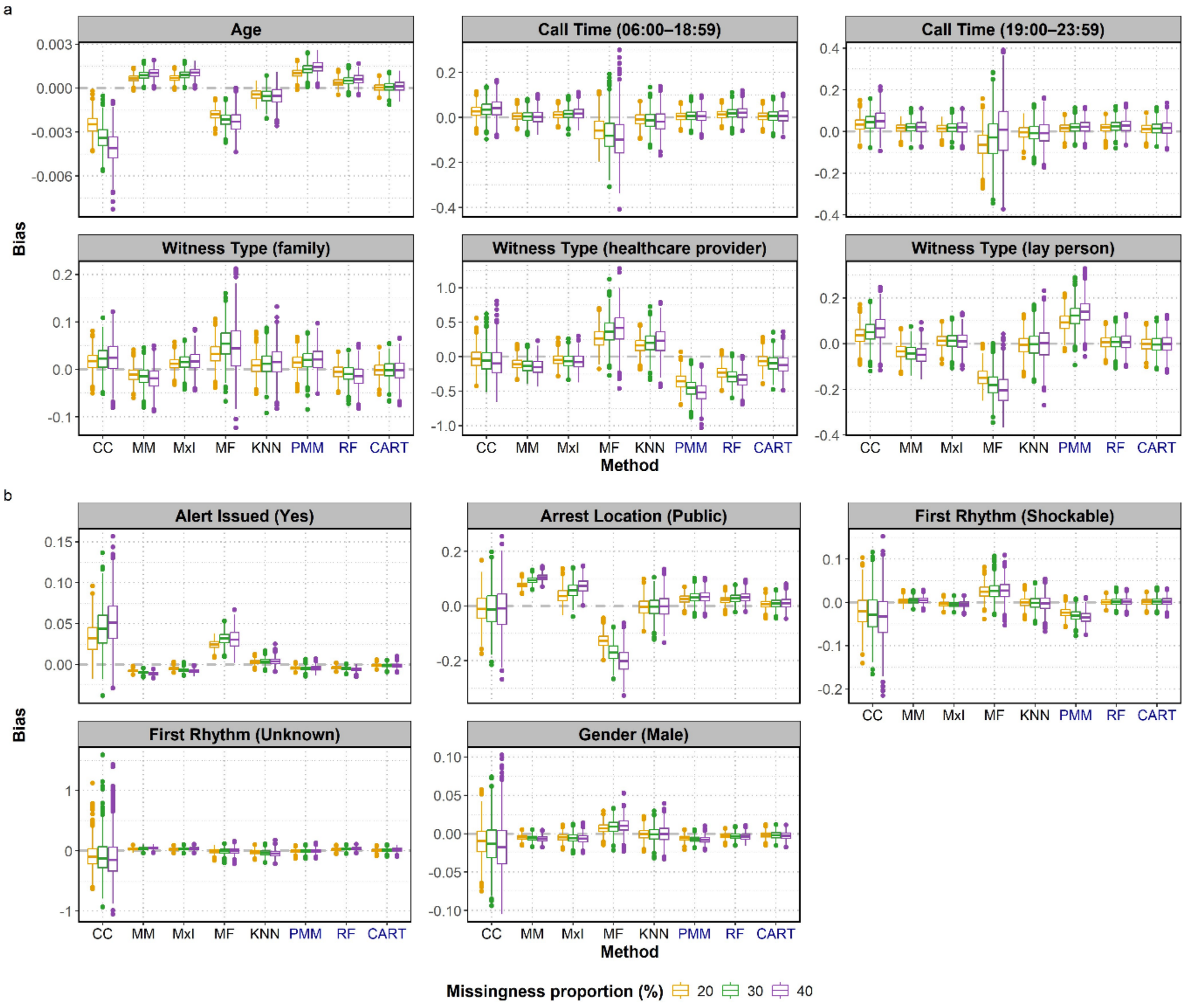


**Figure S1**. Bias for each coefficient in the multivariable logistic regression model predicting BCPR. Results are derived from 1000 simulated datasets based on the OHCA reference dataset (N=13,274), where missingness was artificially introduced at different proportions. Box plots are coloured by proportion of missing data: (a) for covariates with missing values and (b) for covariates with non-missing values. Dashed grey line indicates zero bias. REF: no missing data (reference); CC: complete-case; MM: mean/mode; MxI: missingness-indicator; MF: missForest; KNN: k-Nearest Neighbours; PMM: predictive mean matching; RF: random forest; CART: classification and regression trees.

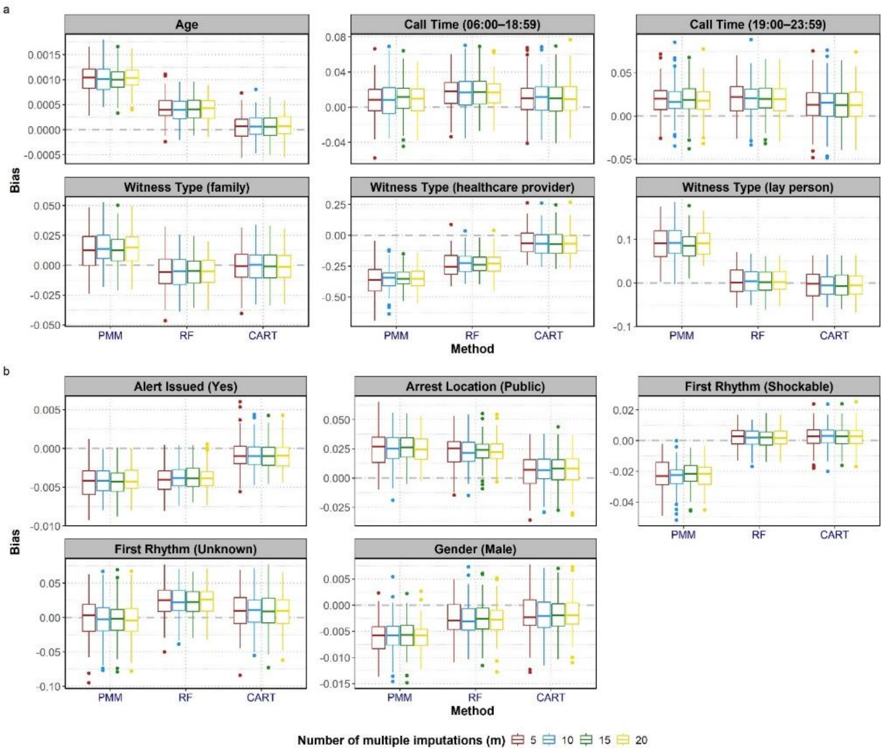


**Figure S2**. Bias for each coefficient in the multivariable logistic regression model predicting BCPR. Results are derived from 100 simulated datasets based on the OHCA reference dataset (N=13,274), where 20% missingness was artificially introduced. Boxplots are coloured by the number of imputations (m): (a) for covariates with missing values and (b) for covariates with non-missing values. Dashed grey line indicates zero bias. PMM: predictive mean matching; RF: random forest; CART: classification and regression trees.

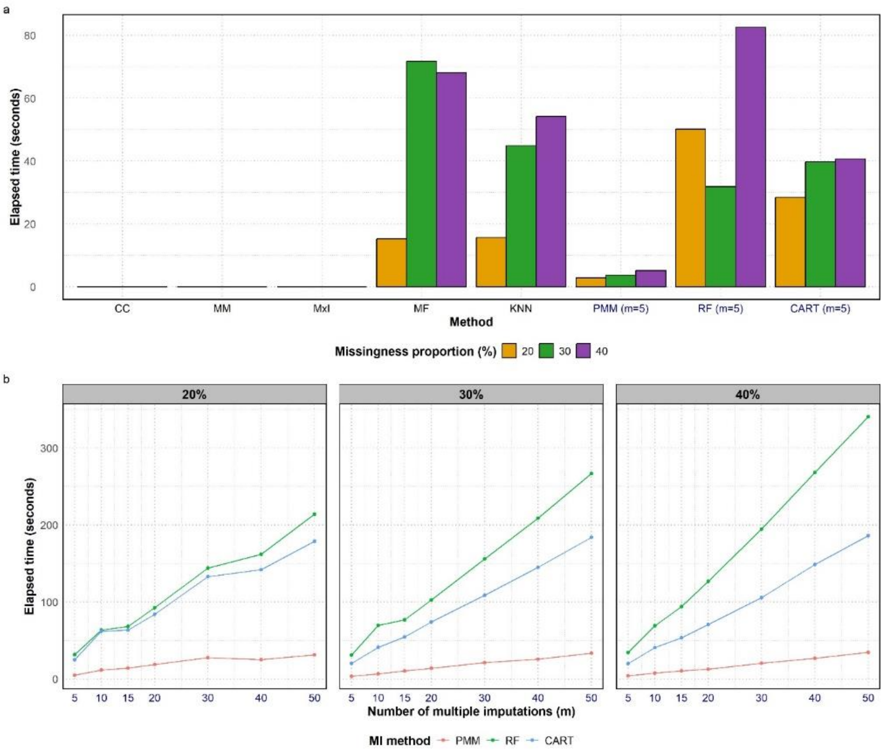


**Figure S3.** Computational time (seconds) for various imputation methods using a randomly selected amputed dataset: (a) imputation time for single imputation methods (CC, MM, MxI, MF, KNN) and multiple imputation methods (PMM, RF, CART) with a fixed number of imputations (m=5). The bars are coloured by missingness proportions (20%, 30%, 40%); (b) imputation time for MI methods as m increases from 5 to 20, faceted by missingness proportions. CC: complete-case; MM: mean/mode; MxI: missingness-indicator; MF: missForest; KNN: k-Nearest Neighbours; PMM: predictive mean matching; RF: random forest; CART: classification and regression trees.

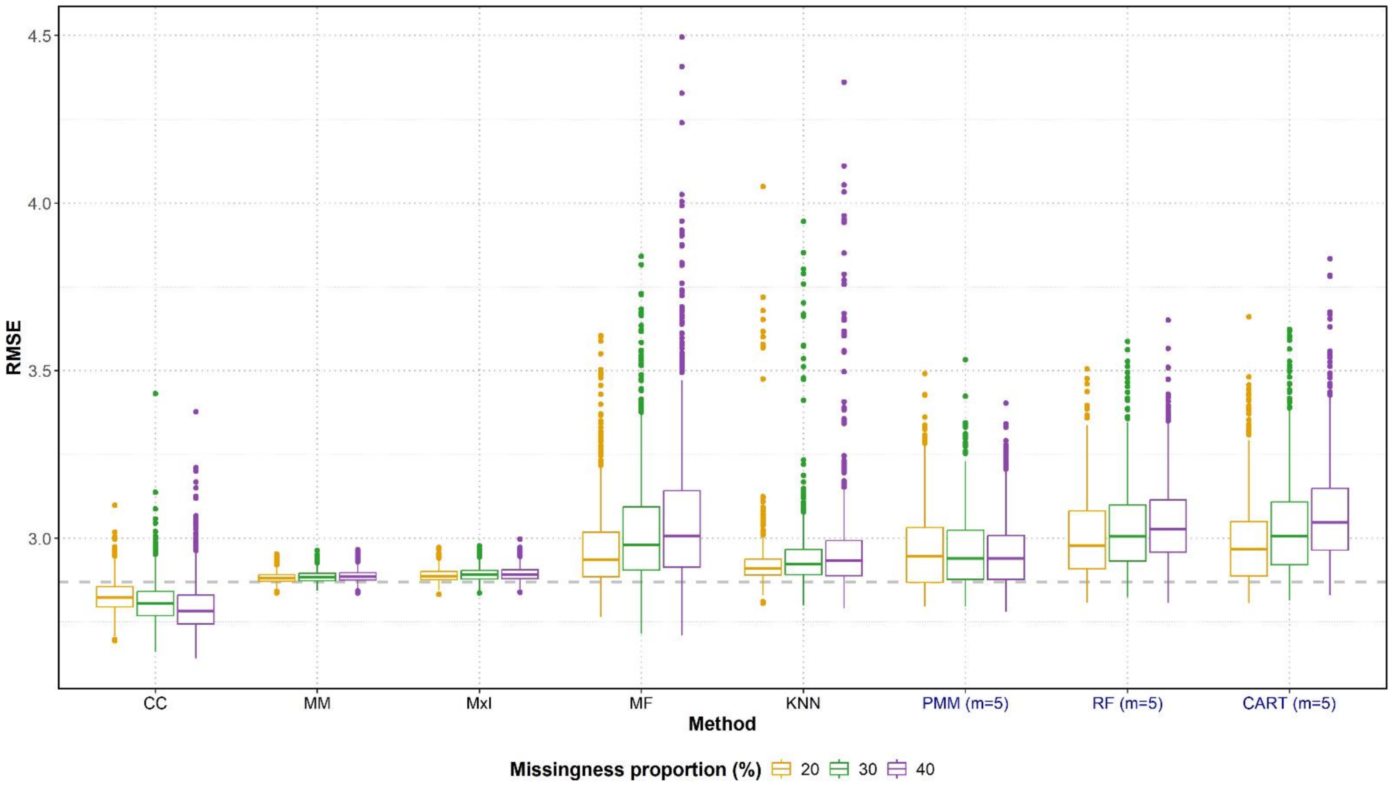


**Figure S4**. RMSE for each model across 1000 simulations grouped by method and coloured by proportion of missing data. Dashed grey line indicates the RMSE from the model based on the reference dataset. CC: complete-case; MM: mean/mode; MxI: missingness-indicator; MF: missForest; KNN: k-Nearest Neighbours; PMM: predictive mean matching; RF: random forest; CART: classification and regression trees.

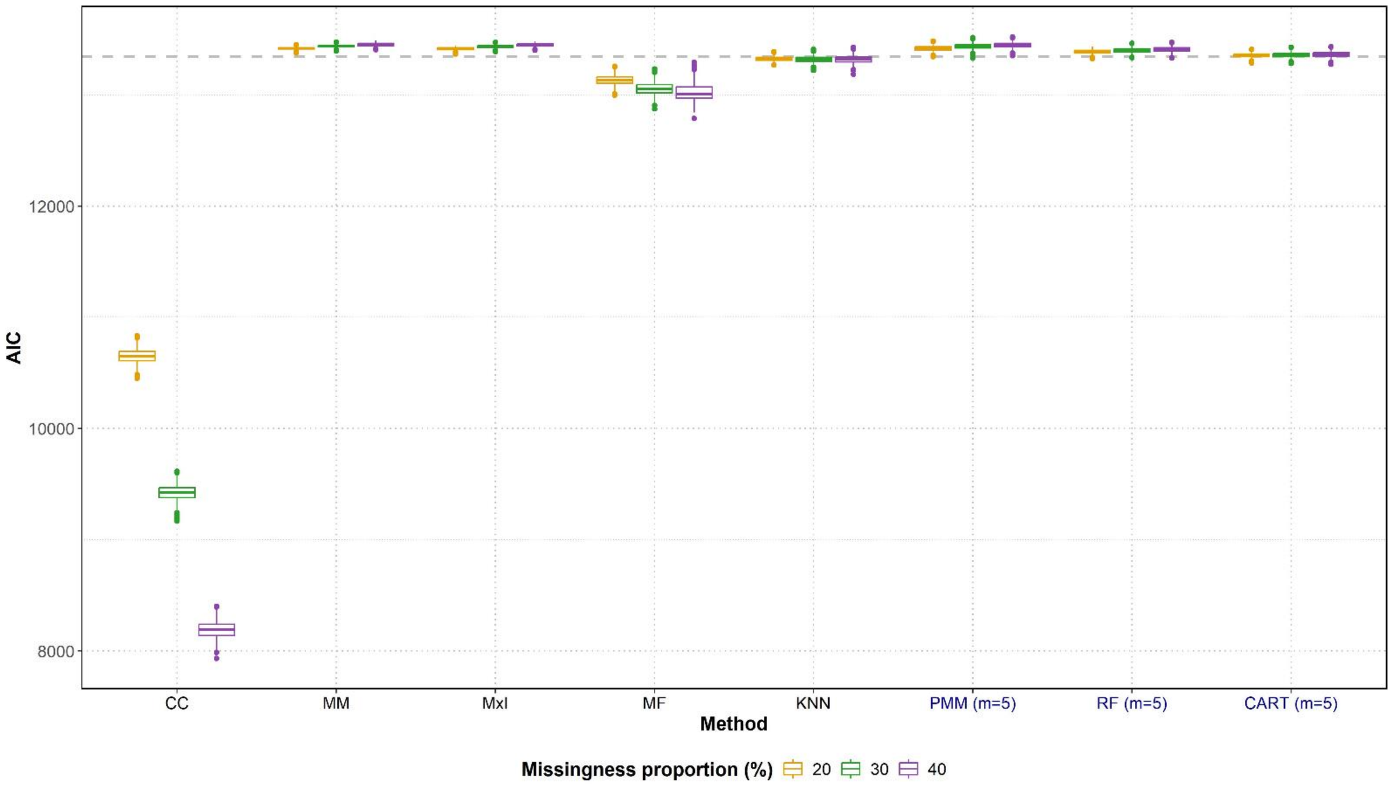


**Figure S5.** AIC for each model across 1000 simulations grouped by method and coloured by proportion of missing data. Dashed grey line indicates the AIC from the full model based on the reference dataset. CC: complete-case; MM: mean/mode; MxI: missingness-indicator; MF: missForest; KNN: k-Nearest Neighbours; PMM: predictive mean matching; RF: random forest; CART: classification and regression trees.

**Table S1.** Multivariable logistic regression analysis of probability of receiving BCPR. Results are derived from 1000 simulated datasets based on the OHCA reference dataset (N=13,274), where missingness was artificially introduced at different proportions (20%, 30% and 40%). Mean β coefficients, empirical standard errors, model-based standard errors and coverage of 95% CI for each covariate. CC: complete-case; MM: mean/mode; MxI: missingness-indicator; MF: missForest; KNN: k-Nearest Neighbours; PMM: predictive mean matching; RF: random forest; CART: classification and regression trees.

| Covariate | Method | 20% missingness proportion | | | | 30% missingness proportion | | | | 40% missingness proportion | | | |
|---|---|---|---|---|---|---|---|---|---|---|---|---|---|
| | | **Mean β coefficient** | **Empirical SE** | **Model-based SE** | **Coverage of 95% CI** | **Mean β coefficient** | **Empirical SE** | **Model-based SE** | **Coverage of 95% CI** | **Mean β coefficient** | **Empirical SE** | **Model-based SE** | **Coverage of 95% CI** |
| Alert issued | REF | 2.43 | *NA* | 0.047 | 100% | 2.43 | *NA* | 0.047 | 100% | 2.43 | *NA* | 0.047 | 100% |
| | CC | 2.46 | 0.019 | 0.051 | 100% | 2.47 | 0.025 | 0.054 | 99.30% | 2.48 | 0.030 | 0.057 | 98% |
| | MM | 2.42 | 0.001 | 0.047 | 100% | 2.42 | 0.002 | 0.047 | 100% | 2.42 | 0.002 | 0.047 | 100% |
| | MxI | 2.42 | 0.002 | 0.047 | 100% | 2.42 | 0.002 | 0.047 | 100% | 2.42 | 0.002 | 0.047 | 100% |
| | MF | 2.45 | 0.005 | 0.047 | 100% | 2.46 | 0.007 | 0.047 | 100% | 2.46 | 0.011 | 0.047 | 100% |
| | KNN | 2.43 | 0.003 | 0.047 | 100% | 2.43 | 0.004 | 0.047 | 100% | 2.43 | 0.005 | 0.047 | 100% |
| | PMM | 2.42 | 0.002 | 0.047 | 100% | 2.42 | 0.003 | 0.047 | 100% | 2.42 | 0.003 | 0.047 | 100% |
| | RF | 2.42 | 0.002 | 0.047 | 100% | 2.42 | 0.002 | 0.047 | 100% | 2.42 | 0.002 | 0.047 | 100% |
| | CART | 2.43 | 0.002 | 0.047 | 100% | 2.43 | 0.002 | 0.047 | 100% | 2.43 | 0.003 | 0.047 | 100% |
| Male | REF | −0.11 | *NA* | 0.047 | 100% | −0.11 | *NA* | 0.047 | 100% | −0.11 | *NA* | 0.047 | 100% |
| | CC | −0.12 | 0.02 | 0.051 | 100% | -0.13 | 0.026 | 0.054 | 100% | -0.13 | 0.033 | 0.058 | 100% |
| | MM | −0.12 | 0.003 | 0.047 | 100% | -0.12 | 0.004 | 0.046 | 100% | -0.12 | 0.004 | 0.046 | 100% |
| | MxI | −0.12 | 0.005 | 0.047 | 100% | -0.12 | 0.006 | 0.047 | 100% | -0.12 | 0.006 | 0.047 | 100% |
| | MF | −0.11 | 0.007 | 0.047 | 100% | -0.10 | 0.008 | 0.047 | 100% | -0.10 | 0.009 | 0.047 | 100% |
| | KNN | −0.11 | 0.007 | 0.047 | 100% | -0.11 | 0.009 | 0.047 | 100% | -0.11 | 0.011 | 0.047 | 100% |
| | PMM | -0.12 | 0.004 | 0.047 | 100% | -0.12 | 0.004 | 0.047 | 100% | -0.12 | 0.005 | 0.047 | 100% |
| | RF | -0.12 | 0.004 | 0.047 | 100% | -0.12 | 0.004 | 0.047 | 100% | -0.12 | 0.004 | 0.047 | 100% |
| | CART | -0.12 | 0.004 | 0.047 | 100% | -0.12 | 0.005 | 0.047 | 100% | -0.12 | 0.005 | 0.047 | 100% |
| Age | REF | 0.0015 | *NA* | 0.001 | 100% | 0.0015 | *NA* | 0.001 | 100% | 0.0015 | *NA* | 0.001 | 100% |
| | CC | −0.001 | 0.0006 | 0.002 | 79.90% | -0.0019 | 0.0008 | 0.002 | 42.10% | -0.0026 | 0.0010 | 0.002 | 29.50% |
| | MM | 0.0022 | 0.0002 | 0.001 | 100% | 0.0024 | 0.0003 | 0.001 | 100% | 0.0025 | 0.0003 | 0.001 | 100% |
| | MxI | 0.0022 | 0.0002 | 0.001 | 100% | 0.0024 | 0.0003 | 0.001 | 100% | 0.0026 | 0.0003 | 0.001 | 100% |
| | MF | −0.0003 | 0.0004 | 0.001 | 99.60% | -0.0007 | 0.0005 | 0.001 | 89% | -0.0008 | 0.0007 | 0.001 | 76% |
| | KNN | 0.0011 | 0.0003 | 0.001 | 100% | 0.001 | 0.0005 | 0.001 | 100% | 0.001 | 0.0006 | 0.001 | 100% |
| | PMM | 0.0025 | 0.0003 | 0.001 | 100% | 0.0028 | 0.0004 | 0.001 | 100% | 0.003 | 0.0004 | 0.002 | 100% |
| | RF | 0.0019 | 0.0003 | 0.001 | 100% | 0.002 | 0.0003 | 0.001 | 100% | 0.0021 | 0.0004 | 0.001 | 100% |

| | | | | | | | | | | | | | |
|---|---|---|---|---|---|---|---|---|---|---|---|---|---|
| | CART | 0.0016 | 0.0003 | 0.001 | 100% | 0.0016 | 0.0003 | 0.001 | 100% | 0.0016 | 0.0004 | 0.001 | 100% |
| Witness type (reference: not witnessed) | | | | | | | | | | | | | |
| Bystander - family | REF | 0.17 | NA | 0.05 | 100% | 0.17 | NA | 0.05 | 100% | 0.17 | NA | 0.05 | 100% |
| | CC | 0.18 | 0.02 | 0.055 | 100% | 0.19 | 0.026 | 0.057 | 100% | 0.19 | 0.033 | 0.061 | 99.90% |
| | MM | 0.16 | 0.015 | 0.051 | 100% | 0.15 | 0.019 | 0.051 | 100% | 0.15 | 0.022 | 0.052 | 100% |
| | MxI | 0.18 | 0.013 | 0.052 | 100% | 0.18 | 0.016 | 0.053 | 100% | 0.19 | 0.020 | 0.054 | 100% |
| | MF | 0.20 | 0.023 | 0.051 | 99.90% | 0.22 | 0.033 | 0.051 | 93.20% | 0.21 | 0.055 | 0.052 | 84.90% |
| | KNN | 0.18 | 0.019 | 0.05 | 100% | 0.18 | 0.024 | 0.05 | 100% | 0.18 | 0.031 | 0.05 | 99.70% |
| | PMM | 0.18 | 0.017 | 0.056 | 100% | 0.19 | 0.021 | 0.06 | 100% | 0.19 | 0.026 | 0.064 | 100% |
| | RF | 0.16 | 0.014 | 0.053 | 100% | 0.16 | 0.018 | 0.054 | 100% | 0.15 | 0.021 | 0.055 | 100% |
| | CART | 0.17 | 0.015 | 0.052 | 100% | 0.17 | 0.019 | 0.053 | 100% | 0.17 | 0.023 | 0.054 | 100% |
| Bystander - healthcare provider | REF | 2.16 | NA | 0.166 | 100% | 2.16 | NA | 0.166 | 100% | 2.16 | NA | 0.166 | 100% |
| | CC | 2.13 | 0.141 | 0.22 | 99.60% | 2.10 | 0.178 | 0.252 | 99.10% | 2.07 | 0.216 | 0.287 | 98.10% |
| | MM | 2.05 | 0.082 | 0.184 | 100% | 2.03 | 0.097 | 0.191 | 99.40% | 2.01 | 0.108 | 0.196 | 99% |
| | MxI | 2.11 | 0.081 | 0.185 | 100% | 2.10 | 0.096 | 0.193 | 100% | 2.09 | 0.108 | 0.198 | 99.90% |
| | MF | 2.42 | 0.144 | 0.153 | 60.90% | 2.52 | 0.185 | 0.152 | 34.70% | 2.57 | 0.231 | 0.152 | 28.30% |
| | KNN | 2.32 | 0.11 | 0.178 | 96.80% | 2.36 | 0.148 | 0.181 | 88.30% | 2.37 | 0.195 | 0.184 | 78.60% |
| | PMM | 1.80 | 0.116 | 0.259 | 77% | 1.70 | 0.127 | 0.282 | 62% | 1.64 | 0.140 | 0.297 | 55.70% |
| | RF | 1.93 | 0.09 | 0.209 | 94.30% | 1.87 | 0.104 | 0.226 | 85.20% | 1.82 | 0.113 | 0.236 | 77.20% |
| | CART | 2.09 | 0.099 | 0.187 | 99.60% | 2.07 | 0.117 | 0.194 | 99.20% | 2.04 | 0.131 | 0.201 | 97.30% |
| Bystander - lay person | REF | −0.11 | NA | 0.067 | 100% | −0.11 | NA | 0.067 | 100% | −0.11 | NA | 0.067 | 100% |
| | CC | −0.07 | 0.04 | 0.08 | 99.90% | -0.06 | 0.047 | 0.087 | 99.60% | -0.04 | 0.059 | 0.095 | 97.50% |
| | MM | −0.14 | 0.033 | 0.068 | 100% | -0.15 | 0.036 | 0.069 | 99.80% | -0.16 | 0.040 | 0.071 | 98.80% |
| | MxI | −0.1 | 0.028 | 0.071 | 100% | -0.10 | 0.034 | 0.073 | 100% | -0.10 | 0.038 | 0.074 | 100% |
| | MF | −0.26 | 0.04 | 0.069 | 36% | -0.29 | 0.052 | 0.07 | 21.10% | -0.31 | 0.068 | 0.071 | 18.20% |
| | KNN | −0.11 | 0.044 | 0.068 | 99.70% | -0.11 | 0.055 | 0.068 | 98.50% | -0.11 | 0.071 | 0.068 | 94.40% |
| | PMM | -0.01 | 0.04 | 0.099 | 96.90% | 0.01 | 0.051 | 0.11 | 91.10% | 0.03 | 0.057 | 0.116 | 84.10% |
| | RF | -0.10 | 0.028 | 0.074 | 100% | -0.10 | 0.034 | 0.076 | 100% | -0.10 | 0.038 | 0.078 | 100% |
| | CART | -0.11 | 0.03 | 0.072 | 100% | -0.11 | 0.037 | 0.074 | 100% | -0.11 | 0.041 | 0.075 | 100% |
| Call time (reference: 00:00–05:59) | | | | | | | | | | | | | |
| 06:00–18:59 | REF | 0.011 | NA | 0.062 | 100% | 0.011 | NA | 0.062 | 100% | 0.011 | NA | 0.062 | 100% |
| | CC | 0.039 | 0.027 | 0.069 | 100% | 0.05 | 0.034 | 0.073 | 100% | 0.05 | 0.043 | 0.078 | 99.50% |

| | | | | | | | | | | | | | |
|---|---|---|---|---|---|---|---|---|---|---|---|---|---|
| | MM | 0.017 | 0.02 | 0.064 | 100% | 0.02 | 0.024 | 0.065 | 100% | 0.01 | 0.028 | 0.066 | 100% |
| | MxI | 0.023 | 0.018 | 0.064 | 100% | 0.03 | 0.023 | 0.066 | 100% | 0.03 | 0.027 | 0.067 | 100% |
| | MF | −0.045 | 0.054 | 0.06 | 86.30% | -0.07 | 0.075 | 0.059 | 68.70% | -0.08 | 0.099 | 0.059 | 55.90% |
| | KNN | 0.003 | 0.028 | 0.063 | 100% | 0.00 | 0.038 | 0.063 | 99.60% | -0.01 | 0.049 | 0.063 | 98.30% |
| | PMM | 0.016 | 0.02 | 0.066 | 100% | 0.02 | 0.025 | 0.068 | 100% | 0.02 | 0.029 | 0.07 | 100% |
| | RF | 0.024 | 0.02 | 0.065 | 100% | 0.03 | 0.024 | 0.066 | 100% | 0.03 | 0.029 | 0.068 | 100% |
| | CART | 0.016 | 0.022 | 0.065 | 100% | 0.02 | 0.028 | 0.067 | 100% | 0.02 | 0.031 | 0.069 | 100% |
| 19:00–23:59 | REF | −0.063 | *NA* | 0.073 | 100% | −0.063 | *NA* | 0.073 | 100% | −0.063 | *NA* | 0.073 | 100% |
| | CC | −0.028 | 0.033 | 0.081 | 100% | -0.02 | 0.041 | 0.086 | 100% | -0.01 | 0.052 | 0.092 | 99.40% |
| | MM | −0.046 | 0.021 | 0.076 | 100% | -0.04 | 0.026 | 0.078 | 100% | -0.04 | 0.031 | 0.079 | 100% |
| | MxI | −0.048 | 0.021 | 0.076 | 100% | -0.04 | 0.026 | 0.078 | 100% | -0.04 | 0.031 | 0.079 | 100% |
| | MF | −0.124 | 0.066 | 0.072 | 88.40% | -0.10 | 0.103 | 0.071 | 79.60% | -0.06 | 0.134 | 0.071 | 69.30% |
| | KNN | −0.066 | 0.033 | 0.074 | 100% | -0.07 | 0.044 | 0.075 | 99.90% | -0.07 | 0.057 | 0.075 | 99% |
| | PMM | -0.047 | 0.022 | 0.077 | 100% | -0.04 | 0.027 | 0.079 | 100% | -0.04 | 0.030 | 0.081 | 100% |
| | RF | -0.044 | 0.023 | 0.077 | 100% | -0.04 | 0.028 | 0.078 | 100% | -0.04 | 0.033 | 0.08 | 100% |
| | CART | -0.051 | 0.025 | 0.077 | 100% | -0.05 | 0.031 | 0.079 | 100% | -0.05 | 0.035 | 0.081 | 100% |
| Public arrest location | REF | 1.03 | *NA* | 0.061 | 100% | 1.03 | *NA* | 0.061 | 100% | 1.03 | *NA* | 0.061 | 100% |
| | CC | 1.03 | 0.052 | 0.078 | 99.70% | 1.02 | 0.068 | 0.09 | 99.60% | 1.02 | 0.084 | 0.104 | 99% |
| | MM | 1.11 | 0.01 | 0.059 | 99.90% | 1.13 | 0.011 | 0.059 | 96.90% | 1.14 | 0.012 | 0.058 | 78.50% |
| | MxI | 1.07 | 0.027 | 0.065 | 99.80% | 1.09 | 0.028 | 0.064 | 99.50% | 1.11 | 0.026 | 0.063 | 97.30% |
| | MF | 0.91 | 0.024 | 0.065 | 51.80% | 0.87 | 0.033 | 0.067 | 13.40% | 0.83 | 0.044 | 0.068 | 7.30% |
| | KNN | 1.03 | 0.031 | 0.066 | 100% | 1.03 | 0.036 | 0.067 | 100% | 1.03 | 0.041 | 0.066 | 99.80% |
| | PMM | 1.06 | 0.016 | 0.066 | 100% | 1.07 | 0.020 | 0.069 | 100% | 1.07 | 0.023 | 0.071 | 100% |
| | RF | 1.06 | 0.014 | 0.063 | 100% | 1.06 | 0.017 | 0.064 | 100% | 1.07 | 0.018 | 0.065 | 100% |
| | CART | 1.04 | 0.015 | 0.062 | 100% | 1.04 | 0.018 | 0.063 | 100% | 1.04 | 0.020 | 0.063 | 100% |
| First rhythm (reference: unshockable) | | | | | | | | | | | | | |
| Shockable | REF | 0.58 | *NA* | 0.066 | 100% | 0.58 | *NA* | 0.066 | 100% | 0.58 | *NA* | 0.066 | 100% |
| | CC | 0.56 | 0.036 | 0.077 | 100% | 0.55 | 0.045 | 0.083 | 99.90% | 0.54 | 0.054 | 0.09 | 99.60% |
| | MM | 0.58 | 0.007 | 0.065 | 100% | 0.58 | 0.007 | 0.065 | 100% | 0.58 | 0.007 | 0.065 | 100% |
| | MxI | 0.57 | 0.006 | 0.065 | 100% | 0.57 | 0.007 | 0.065 | 100% | 0.57 | 0.007 | 0.065 | 100% |
| | MF | 0.60 | 0.016 | 0.067 | 100% | 0.60 | 0.020 | 0.067 | 100% | 0.60 | 0.022 | 0.067 | 100% |
| | KNN | 0.58 | 0.012 | 0.066 | 100% | 0.58 | 0.015 | 0.066 | 100% | 0.58 | 0.019 | 0.066 | 100% |
| | PMM | 0.55 | 0.010 | 0.068 | 100% | 0.55 | 0.012 | 0.069 | 100% | 0.54 | 0.013 | 0.069 | 100% |

| | | | | | | | | | | | | | |
|---|---|---|---|---|---|---|---|---|---|---|---|---|---|
| | RF | 0.58 | 0.007 | 0.066 | 100% | 0.58 | 0.008 | 0.066 | 100% | 0.58 | 0.009 | 0.066 | 100% |
| | CART | 0.58 | 0.008 | 0.066 | 100% | 0.58 | 0.009 | 0.066 | 100% | 0.58 | 0.010 | 0.066 | 100% |
| Unknown | REF | 2.03 | *NA* | 0.385 | 100% | 2.03 | *NA* | 0.385 | 100% | 2.03 | *NA* | 0.385 | 100% |
| | CC | 1.95 | 0.206 | 0.444 | 100% | 1.93 | 0.267 | 0.479 | 99.90% | 1.91 | 0.332 | 0.522 | 99.80% |
| | MM | 2.06 | 0.023 | 0.384 | 100% | 2.07 | 0.024 | 0.384 | 100% | 2.07 | 0.025 | 0.383 | 100% |
| | MxI | 2.05 | 0.022 | 0.384 | 100% | 2.06 | 0.023 | 0.384 | 100% | 2.07 | 0.024 | 0.383 | 100% |
| | MF | 2.02 | 0.038 | 0.388 | 100% | 2.02 | 0.046 | 0.388 | 100% | 2.02 | 0.051 | 0.389 | 100% |
| | KNN | 2.00 | 0.038 | 0.387 | 100% | 1.99 | 0.047 | 0.387 | 100% | 1.98 | 0.057 | 0.387 | 100% |
| | PMM | 2.02 | 0.029 | 0.386 | 100% | 2.02 | 0.035 | 0.387 | 100% | 2.02 | 0.037 | 0.387 | 100% |
| | RF | 2.05 | 0.022 | 0.385 | 100% | 2.06 | 0.024 | 0.385 | 100% | 2.06 | 0.025 | 0.385 | 100% |
| | CART | 2.04 | 0.027 | 0.386 | 100% | 2.04 | 0.030 | 0.386 | 100% | 2.04 | 0.033 | 0.386 | 100% |

SE, standard errors; CI, confidence interval
